# Exploring professional identity and its predictors in health profession students and healthcare practitioners in Saudi Arabia

**DOI:** 10.1371/journal.pone.0299356

**Published:** 2024-05-31

**Authors:** Walaa A. Mumena, Bandar A. Alsharif, Abdulaziz M. Bakhsh, Waleed H. Mahallawi

**Affiliations:** 1 Clinical Nutrition Department, College of Applied Medical Sciences, Taibah University, Madinah, Saudi Arabia; 2 Department of Education, College of Arabic Language and Humanities Studies, Islamic University of Madinah, Madinah, Saudi Arabia; 3 Urology Department, College of Medicine, Taibah University, Madinah, Saudi Arabia; 4 Medical Laboratory Technology Department, College of Applied Medical Sciences, Taibah University, Madinah, Saudi Arabia; Alexandria University Faculty of Nursing, EGYPT

## Abstract

The government of Saudi Arabia is making significant efforts to improve the quality of health education and healthcare services. Professional identity has been linked to the quality of healthcare services provided by practitioners, however, data concerning the professional identity of health profession students (HPS) and healthcare practitioners (HCP) are still lacking in Saudi Arabia. The current study aimed to assess the level of professional identity in HPS and HCP in Saudi Arabia and to investigate its predictors. Cross-sectional data were collected from 185 HPS and 219 HCP in Saudi Arabia using river sampling technique. Data related to the sample characteristics were collected; an adapted version of the Macleod Clark Professional Identity Scale was utilized to collect data about the level of professional identity. Total score of professional identity was later calculated for each participant. Median professional identity scores for HPS and HCP were 38.0 (34.0–41.0) and 41.0 (37.0–43.0), respectively, out of 45. Significantly higher median professional identity score was found among HCP as compared to HPS (*p* <0.001). Data obtained from the multiple linear regression analysis, using the backward elimination method technique indicated that only working status (HPS vs. HCP) significantly predicted the professional identity score in all models performed. In conclusion, high levels of professional identity were reported among HCP and HPS in Saudi Arabia. Changes related to professional identity should be monitored in public and private educational and healthcare organizations to enhance the quality of healthcare services provided in the country.

## Introduction

Professional identity is a term used to describe the attitudes, values, knowledge, beliefs, and skills shared with others within a professional group [1, 2]. It has been noted that professional identity develops continuously, starting from experiences gained informally in life and formally in classrooms. These experiences continue to evolve into the work life of individuals that can be influenced by a number of factors including skills and knowledge [3–6]. Research has shown that all educational experiences outside of the classroom can influence students’ level of professional identity [4, 7, 8]. Therefore, research has focused on assessing the professional identity of students, particularly students enrolled in the healthcare field [1, 2, 6, 9].

Professional identity is an important factor that can influence the safety and effectiveness of clinical practice of all healthcare professions [3]. It has been suggested that professional identity is positively linked to level of satisfaction and commitment to professional standards and guidelines [10–12]. Additionally, high levels of professional identity can result in better communication between healthcare team and patients and strong sense of moral and ethics in regard to decision-making [13].

The formation of professional identity is very challenging and should be planned considering several factors related to the student (e.g., students’ circumstances, values, and attributes) and factors related to the program (e.g., learning environment and curriculum) [6, 14]. Therefore, the Ministry of Education and Ministry of Health in Saudi Arabia are working extensively with academic, healthcare, and other institutions nationally and internationally to improve the quality of education and training provided to students and workers through different strategic plans one of which is the privatization of the education and healthcare systems in the country [15, 16]. It is important for health profession students (HPS) to have a high-level professional identity because it can influence the recognition, expression, and performance of professionals and prioritization of their roles as future healthcare practitioners (HCP) [17, 18].

It has been recommended to assess and monitor the level of professional identity of students to enhance the confidence and performance of future HCP [9, 19]. However, research exploring the level of professional identity among HPS and HCP are still lacking in the Arab world, including Saudi Arabia. Thus, we aim in the current study to assess the level of professional identity in HPS and HCP in Saudi Arabia and to investigate its predictors.

## Materials and methods

### Design, setting, and participants

A cross-sectional design was used to recruit HPS and HCP in Saudi Arabia between May and June 2022. Participants not enrolled in health program or working in the healthcare field were excluded. The minimum number of participants needed for this study was 184 HPS and 184 HCP based on expected number of healthcare students/workers in Saudi Arabia of 300,000, expected frequency of high levels of professional identity among 40% of the population, and 5% margin of error. Sample size calculations were done using Epi Info^TM^ (Epi Info 7.2.4.0, CDC, Atlanta).

Ethical certificate to conduct this study was obtained from the Research Ethics Committee of the College of Applied Medical Sciences, Taibah University [certificate number: 2022/140/104/MLT]. All participants provided electronic written consent to be part of the study before collecting their data via the online survey.

### Data collection

Data were collected from HPS and HCP in Saudi Arabia using river sampling technique, where link of the survey was shared with HPS and HCP via multiple social media applications (WhatsApp and Twitter). Deans of health colleges in universities around Saudi Arabia were contacted to assess in the distribution of the survey to students, interns, graduates, and faculty members working part-time in healthcare intuitions. Demographic data and information concerning professional identity were gathered. No personal information or contact numbers were collected in this study and participants recruited in this study are not identifiable.

### Sociodemographics data

Information concerning gender, nationality, and marital status was collected from all participants included in this study. Data related to specialty (Applied Medical Sciences; Medicine/Dentistry; Nursing; other specialties), institution (public; private), year of study (year 1–2; year 3–4; year 5–6; internship), and academic performance (cumulative GPA) were collected from all HPS. Data related to specialty (Applied Medical Sciences; Medicine/Dentistry; Nursing; other specialties), institution (public; private), age group (20–30 years; 31–40 years; 41–50 years; > 50 years), qualification (diploma; bachelor’s degree; master’s degree; Ph.D./fellowship/subspecialty), job rank (technician; specialist/senior specialist; physician/resident; consultant), and year of experience (1–5 years; 6–10 years; 11–15 years; 16–20 years; > 20 years) were collected from HCP.

### Assessment of professional identity

The Macleod Clark Professional Identity Scale (MCPIS) was used to assess the professional identity of HPS, which consists of nine items (MCPIS-9) [9]. The MCPIS has been frequently used to assess professional identity and found to be the most appropriate tool to assess of professional identity in a range of health professionals [3]. An adapted version of the tool was used to assess the professional identity of HCP. Modifications were done to ensure the target population is HCP. Expert and content validity were conducted to ensure clarity of the items included in the modified version of the tool. Items included are: 1) I feel like a member of my profession, in which I work; 2) I feel that I have strong relationships with practitioners in my profession; 3) I feel ashamed to acknowledge that I belong to my profession; 4) I find myself providing excuses to belong to my profession; 5) I try to hide that I am part of this profession; 6) I am glad to belong to this profession; 7) I can identify positively with members of this profession; 8) Being a member of this profession is important to me; 9) I feel I share characteristics with other members of the profession.

Responses to items were reported using Likert Scale as follows: “Strongly Agree”, “Agree”, “Neutral”, “Disagree”, and “Strongly Disagree.” Items 1,2,6,7,8 and 9 were coded as 5, 4, 3, 2, and 1, respectively, while for items 3,4, and 5 responses were coded 1, 2, 3, 4, and 5, respectively. A total score of the 9 items was later calculated with maximum of 45 points. The internal reliability of this scale among HPS and HCP was good for both groups (Cronbach’s alpha of 0.81 and 0.77, respectively).

### Statistical analysis

We analyzed the collected data using IBM SPSS Statistics for Windows (Version 20.0. Armonk, NY, USA: IBM Corp). Descriptive data for categorical variables were presented as frequencies (percentages), and descriptive data for continuous variables were presented as median and interquartile range. The Shapiro–Wilk test was used to assess the normality of the professional identity distribution and results show that distribution was skewed (*p*< 0.05). To compare the median score of the professional identity between HPS and HCP, the Mann–Whitney U test was used. Additionally, the Mann–Whitney U test and Kruskal–Wallis test were used to compare the medians across the different groups (e.g., gender, nationality, education level). Pairwise comparisons were conducted to further investigated findings of the Kruskal–Wallis tests performed. Multiple linear regression analysis, using the backward elimination method technique, was conducted to explore predictors of professional identity (dependent variable) and sample characteristics (independent variables). All tests used were two-tailed with a significance level of 95%.

## Results

### Sample characteristics

A total of 185 HPS and 219 HCP were included in this study. Half of the HPS were men (n = 93), whereas 73.5% (n = 161) of the HCP were men. All the HPS were Saudi (n = 185), whereas 81.7% (n = 179) of the HCP were Saudi. The majority of the HPS (96.2%, n = 178) were single, whereas 78.5% (n = 172) of HCP were married. Most of the HPS and HCP specialized in applied medical sciences (68.1% and 42.0%, respectively). Ninety-five percent of the HPS (n = 175) were studying at a public education institution, whereas 83.6% (n = 183) of the HCP reported working at a public institution. Detailed description of the study sample is provided in **Table 1**.

**Table 1:** Sample characteristics.

	Health Profession Students (n = 185)	Healthcare Practitioners (n = 219)	Total (n = 404)
**Gender**			
Male	93 (50.3)	161 (73.5)	254 (62.9)
Female	92 (49.7)	58 (26.5)	150 (37.1)
**Nationality**			
Saudi	185 (100)	179 (81.7)	364 (90.1)
Non-Saudi	0 (0.00)	40 (18.3)	40 (9.90)
**Marital status**			
Single	178 (96.2)	47 (21.5)	225 (55.7)
Married	7 (3.80)	172 (78.5)	179 (44.3)
**Specialization**			
Applied Medical Sciences	126 (68.1)	92 (42.0)	218 (54.0)
Medicine/Dentistry	4 (2.20)	59 (26.9)	63 (15.6)
Nursing	4 (2.20)	19 (8.70)	23 (5.69)
Other specialties	51 (27.6)	49 (22.4)	100 (24.8)
**Institution**			
Public	175 (94.6)	183 (83.6)	358 (88.6)
Private	10 (5.40)	36 (16.4)	46 (11.4)
**Year of study**			
1–2	45 (24.3)		45 (24.3)
3–4	73 (39.5)		73 (39.5)
5–6	41 (22.2)		41 (22.2)
Internship	26 (14.1)		26 (14.1)
**Cumulative GPA** ^**a**^			
Excellent (4.5–5.0)	87 (47.0)		87 (47.0)
Very good (3.75–4.49)	70 (37.8)		70 (37.8)
Good (2.75–3.74)	28 (15.1)		28 (15.1)
**Age group**			
20–30 years		33 (15.1)	33 (15.1)
31–40 years		104 (47.5)	104 (47.5)
41–50 years		47 (21.5)	47 (21.5)
> 50 years		35 (16.0)	35 (16.0)
**Qualification**			
Diploma		52 (23.7)	52 (23.7)
Bachelor’s degree		79 (36.1)	79 (36.1)
Master’s degree		31 (14.2)	31 (14.2)
Ph.D./fellowship/subspecialty		57 (26.0)	57 (26.0)
**Job rank**			
Technician		64 (29.2)	64 (29.2)
Specialist/senior specialist		98 (44.7)	98 (44.7)
Physician/resident		18 (8.20)	18 (8.20)
Consultant		39 (17.8)	39 (17.8)
**Years of experience**			
1–5 years		38 (17.4)	38 (17.4)
6–10 years		60 (27.4)	60 (27.4)
11–15 years		43 (19.6)	43 (19.6)
16–20 years		30 (13.7)	30 (13.7)
> 20 years		48 (21.9)	48 (21.9)

Numbers presented in table are frequencies (%).

^***a***^GPA: grade point average out of 5.0.

### Level of professional identity

Detailed data concerning responses to items included in the MCPIS-9 used among HPS and HCP in Saudi Arabia are provided in **S1 Table**. Median professional identity score was 38.0 (34.0–41.0) among HPS, while median professional identity score was 41.0 (37.0–43.0) among HCP out of 45, which indicate high levels of professional identity (84.4% and 91.1%, respectively). The Mann–Whitney U test was used and result show significantly higher median professional identity score among HCP as compared to HPS (*p* <0.001).

### Association between professional identity and characteristics of the study sample

Data concerning the association between professional identity level and characteristics of HPS and HCP are presented in **Table 2**. Median score of professional identity among HPS was similar across the different groups. Median professional identity score was significantly higher among married HCP compared to their single counterparts (41.0 [38.0–44.0] vs. 40.0 [36.0–42.0], respectively, *p* = 0.024). The median professional identity score was significantly lower among HCP specializing in applied medical sciences compared to those from all other specialties (Medicine/Dentistry: *p* = 0.011; Nursing: *p* = 0.031; other: *p* = 0.013). The median professional identity score was significantly lower among HCP aged 20–30 years compared to HCP aged >50 years (41.0 [37.0–43.0] vs. 43.0 [39.0–44.0], respectively, *p* = 0.016). In addition, the median professional identity score was significantly lower among HCP aged 31–40 years compared to those aged >50 years (40.0 [36.0–43.0] vs. 43.0 [39.0–44.0], respectively, *p* = 0.009). The median professional identity score was significantly lower among practitioners holding a bachelor’s degree compared to those with a Ph.D., fellowship, or subspecialty (40.0 [37.0–42.0] vs. 41.0 [37.5–44.0], respectively, *p* = 0.037). The median professional identity score was significantly lower among HCP holding a bachelor’s degree compared to those with a diploma (40.0 [37.0–42.0] vs. 42.0 [39.0–44.0], respectively, *p* = 0.007). The median professional identity score was significantly lower among HCP holding a master’s degree compared to those with a diploma (40.0 [36.0–43.0] vs. 42.0 [39.0–44.0], respectively, *p* = 0.043). The median professional identity score was significantly higher among HCP with >20 years of work experience compared to those with ≤15 years of work experience (1–5 years: *p* <0.001; 6–10 years: *p* = 0.001; 11–15 years: *p* = 0.020).

**Table 2:** Association between professional identity level and characteristics of health profession students and healthcare practitioners.

	Health Profession Students (n = 185)	Healthcare Practitioners (n = 219)
**Gender** ^**a**^		
Male	38.0 (35.0–41.0)	41.0 (38.0–43.0)
Female	37.5 (34.0–41.0)	39.5 (37.0–43.0)
*p-value*	0.287	0.175
**Year of study** ^**b**^		
1–2	37.0 (34.5–41.0)	
3–4	38.0 (35.0–42.0)	
5–6	39.0 (34.0–41.0)	
Internship	38.5 (34.0–41.3)	
*p-value*	0.637	
**Cumulative GPA** ^**a**^		
Excellent (4.5–5.0)	38.0 (34.0–41.0)	
Very good (3.75–4.49)	38.0 (35.0–41.3)	
Good (2.75–3.74)	37.5 (33.0–40.8)	
*p-value*	0.637	
**Nationality** ^**a**^		
Saudi	38.0 (34.5–41.0)	41.0 (37.0–43.0)
Non-Saudi	-	41.5 (38.0–44.0)
*p-value*	-	0.336
**Marital status** ^**a**^		
Single	38.0 (34.0–41.0)	40.0 (36.0–42.0)
Married	39.0 (38.0–40.0)	41.0 (38.0–44.0)
*p-value*	0.657	0.024*
**Specialization** ^**b**^		
Applied Medical Sciences	38.0 (34.0–41.0)	39.0 (37.0–42.0)
Medicine/Dentistry	40.0 (31.5–41.8)	42.0 (38.0–44.0)
Nursing	38.0 (32.5–41.3)	41.0 (40.0–45.0)
Other specialties	38.0 (35.0–41.0)	42.0 (38.5–43.0)
*p-value*	0.986	0.012*
**Institution** ^**a**^		
Public	38.0 (35.0–41.0)	41.0 (37.0–43.0)
Private	39.0 (33.3–42.5)	41.0 (35.8–43.8)
*p-value*		0.586
**Age group** ^**b**^		
20–30 years		41.0 (37.0–43.0)
31–40 years		40.0 (36.0–43.0)
41–50 years		41.0 (38.0–43.0)
> 50 years		43.0 (39.0–44.0)
*p-value*		0.032*
**Qualification** ^**b**^		
Diploma		42.0 (39.0–44.0)
Bachelor’s degree		40.0 (37.0–42.0)
Master’s degree		40.0 (36.0–43.0)
Ph.D./fellowship/subspecialty		41.0 (37.5–44.0)
*p-value*		0.021*
**Job rank** ^**b**^		
Technician		41.0 (38.3–44.0)
Specialist/senior specialist		40.0 (36.8–42.3)
Physician/resident		39.0 (36.5–43.3)
Consultant		42.0 (38.0–44.0)
*p-value*		0.077
**Years of experience** ^**b**^		
1–5 years		38.5 (36.8–42.0)
6–10 years		40.0 (36.3–42.8)
11–15 years		41.0 (36.0–43.0)
16–20 years		41.0 (37.0–44.0)
> 20 years		43.0 (39.3–44.8)
*p-value*		0.003*

*Significant at the 95% confidence level. ^***a***^GPA: grade point average out of 5.0. The

^**a**^Mann–Whitney U and

^**b**^Kruskal–Wallis tests were used to obtain data presented in table.

Data obtained from the multiple linear regression analysis, using the backward elimination method technique, of predictors of professional identity among HPS and HCP in Saudi Arabia are presented in **Table 3**. Only working status (HPS vs. HCP) significantly predicted the professional identity score in all models obtained.

**Table 3:** Multiple linear regression analysis of predictors of professional identity among health profession students and healthcare practitioners.

	Beta	Standard Error	95% Confidence Interval	*p*-value
**Model 1**				
Working status (health profession students = 1, healthcare practitioners = 2)	1.75	0.73	0.32 to 3.18	0.017*
Gender (male = 1; femele = 2)	-0.40	0.55	-1.47 to 0.67	0.464
Nationality (Saudi = 1; non-Saudi = 1)	0.04	0.85	-1.64 to 1.72	0.961
Marital ststus (single = 1; married = 2)	1.05	0.77	-0.47 to 2.58	0.175
Specialization (Applied Medical Sciences = 1; Medicine = 2; Nursing = 3; other specialties = 4)	0.28	0.19	-0.10 to 0.66	0.148
**Model 2**				
Working status (health profession students = 1, healthcare practitioners = 2)	1.75	0.73	0.33 to 3.18	0.016*
Gender (male = 1; femele = 2)	-0.40	0.54	-1.47 to 0.67	0.463
Marital ststus (single = 1; married = 2)	1.06	0.77	-0.45 to 2.57	0.169
Specialization (Applied Medical Sciences = 1; Medicine = 2; Nursing = 3; other specialties = 4)	0.28	0.19	-0.10 to 0.66	0.143
**Model 3**				
Working status (health profession students = 1, healthcare practitioners = 2)	1.71	0.84	0.29 to 3.13	0.018*
Marital ststus (single = 1; married = 2)	1.23	0.73	-0.21 to 2.66	0.093
Specialization (Applied Medical Sciences = 1; Medicine = 2; Nursing = 3; other specialties = 4)	0.30	0.19	-0.07 to 0.67	0.106
**Model 4**				
Working status (health profession students = 1, healthcare practitioners = 2)	1.72	0.72	0.30 to 3.14	0.018*
Marital ststus (single = 1; married = 2)	1.38	0.73	-0.05 to 2.81	0.058

*Significant at the 95% confidence level.

## Discussion

The government of Saudi Arabia is making significant efforts to improve the quality of education and healthcare system in the country. Professional identity has been linked to the quality of healthcare services provided by practitioners. Yet, data concerning the professional identity of HPS and HCP are still lacking in Saudi Arabia. The current study explored the level of professional identity in HPS and HCP and investigated potential predictors in Saudi Arabia. Findings of this study will serve as benchmark data that can guide decision-makers in the education system pre- and post-privatization of public universities occurring in the country. Results of this study show high levels of professional identity among both HPS and HCP. Significantly higher median professional identity score was reported among HCP as compared to HPS. Our results indicated that only working status (HPS vs. HCP) significantly predicted the professional identity score.

The current study show high levels of professional identity among HPS and HCP in Saudi Arabis with median of over 80% for both groups. A study conducted among nursing students in Iran reported semilar level of professional identity (83.8%) [20]. High levels of professional identity reported in our study among HPS and HCP can reflect the attention gevin to future healthcare workers/staff in the country. In Saudi Arabia, privatization of the healthcare system has been done, whereas the privatization of all public university is being gradually implemented. Thus, assessing the changes occurring with this transformation is essential to ensure the quality of services provided in healthcare organizations at the national level. Additionally, it is important to monitor the professional identity of HPS and HCP to enhance the confidence and performance of HCP as well as the services provided in healthcare organizations in the country.

In the current study, significantly higher median professional identity score was observed among HCP as compared to HPS. Research suggests that skills and experience are important factors that have been linked directly to professional identity of healthcare practitioners, which can explain the higher level of professional identity reported among HCP included in our study [3, 4, 6]. Most healthcare practitioners are expected to have high level of professional identity that is somewhat influenced by their experience. In addition, lower level of professional identity reported among HPS was probably caused by the retention rates of students registered in academic programs temporarily aiming to switch to other healthcare majors (e.g., student enrolled in a nursing program but actually interested to study pharmacy) [9, 19]. In fact, many students typically transfer during their studies to other programs that expected to be a better fit for them, whereas HCP are less common to change career.

Several factors have been found to be associated with professional identity of students. A recent study conducted by Adams et al. indicated that gender, knowledge of profession, previous work experience in healthcare environments, and many other factors are linked to level of professional identity [1]. However, our findings indicated no link between characteristics of HPS and level of professional identity. This findings can be explained by the similar characteristics of HPS in Saudi Arabia which is caused by previous regulations related to the acceptance of students in the majority of Saudi universities. For example, most students accepted are fresh high-school graduates, Saudis, and students who obtained high scores in all standardized tests. However, median professional identity score was significantly lower among HCP specializing in Applied Medical Sciences compared to those from all other specialties. Possibly large proportion of students in this field were intrested in enrolling in other healthcare majors (e.g. Medicine or Dentistry) but they were not accepted, thus most of these students feel less intrested in the field they are studying.

Research exploring approaches that can help in enhancing the level of professional identity is important to be conducted in Saudi Arabia. A recent review that concluded that mentoring, reflection, self-efficacy, goal orientation, professional socialization, and critical thinking are competencies that can help in the development of the professional identity of medical students. These components can be very helpful if included in the curriculum in order to shift it to be effective competency-based curriculum [21]. A study conducted in 2021 among nursing students during their internship period recommended eductors to focus on the professional identity and self-efficacy of students in order to improve their competence [22].

To the best of our knowledge, our study is the first in Saudi Arabia to investigate professional identity among HPS and HCP. The study is limited by the nature of the design used which does not allow the determination of causality and change in patterns. Additionally, due to the sampling method technique used to recruit participants, the generalizability of the study findings might be limited to participants who used social media applications frequently. However, most of the Saudi population, especially young and educated individuals, have Internet access and use social media applications frequently [23, 24].

## Conclusions

High level of professional identity was reported among HPS and HCP in Saudi Arabia. Number of factors found to be associated with the level of professional identity among HCP but not HPS. Educational and healthcare organizations are encouraged to make continues efforts to enhance the level of professional identity among HPS during their studies. Longitudinal research should be directed to explore changes related to professional identity after privatization of all public university in Saudi Arabia is done.

## Supporting information

S1 Checklist STROBE statement—Checklist of items that should be included in reports of observational studies. (DOCX)

S1 Table Items included in the professional identity scale among health profession students and healthcare practitioners in Saudi Arabia. Numbers presented in table are frequencies (%).
(DOCX)
