# Supplementary material for: Exploring professional identity and its predictors in health profession students and healthcare practitioners in Saudi Arabia
Source: PLoS One. 2024 May 31;19(5):e0299356. doi: 10.1371/journal.pone.0299356 (PMC11142680; doi:10.1371/journal.pone.0299356)
Supplement: S1 Checklist — (DOCX) [file pone.0299356.s001.docx]

STROBE Statement—checklist of items that should be included in reports of observational studies

|  | Item No. | Recommendation | Page  No. | Relevant text from manuscript |
| --- | --- | --- | --- | --- |
| **Title and abstract** | 1 | (*a*) Indicate the study’s design with a commonly used term in the title or the abstract | 2 | Cross-sectional data were collected from 185 HPS and 219 HCP in Saudi Arabia using river sampling technique |
|  |  | (*b*) Provide in the abstract an informative and balanced summary of what was done and what was found | 2 | Cross-sectional data were collected from 185 HPS and 219 HCP in Saudi Arabia using river sampling technique. Demographic data were collected and an adapted version of the Macleod Clark Professional Identity Scale was utilized to collect data concerning professional identity. Total score of professional identity was later calculated for each participant. Median professional identity score for HPS was 38.0 (34.0–41.0) and 41.0 (37.0–43.0) for HCP out of 45, which indicate strong levels of professional identity (84.4% and 91.1%, respectively). Significantly higher median professional identity score was found among HCP as compared to HPS (p <0.001). None of the students’ characteristics predicted level of professional identity, whereas age group predicted HCP level of professional identity (Beta=0.87, [95% Confidence Interval: 0.30 to 1.44], specialization (Beta=0.58, [95% Confidence Interval: 0.14 to 1.01], and years of experience (Beta=0.72, [95% Confidence Interval: 0.35 to 1.10]. |
| Introduction | | | |  |
| Background/rationale | 2 | Explain the scientific background and rationale for the investigation being reported | 4 | It has been recommended to assess and monitor the professional identity of students to enhance the confidence and performance of future HCP. However, research exploring the level of professional identity among health professions students (HPS) and HCP are still lacking in the Arab world, including Saudi Arabia. |
| Objectives | 3 | State specific objectives, including any prespecified hypotheses | 4 | we aim in the current study to assess the level of professional identity in HPS and HCP and to investigate its predictors in Saudi Arabia. |
| Methods | | | |  |
| Study design | 4 | Present key elements of study design early in the paper | 4 | A cross-sectional design was used to recruit HPS and HCP in Saudi Arabia. |
| Setting | 5 | Describe the setting, locations, and relevant dates, including periods of recruitment, exposure, follow-up, and data collection | 5 | Data were collected from HPS and HCP in Saudi Arabia using river sampling technique, where link of the survey was shared with HPS and HCP via multiple social media applications (WhatsApp and Twitter). Deans of health colleges in universities around Saudi Arabia were contacted to assess in the distribution of the survey. Demographic data and information concerning professional identity were gathered. |
| Participants | 6 | (*a*) *Cohort study*—Give the eligibility criteria, and the sources and methods of selection of participants. Describe methods of follow-up  *Case-control study*—Give the eligibility criteria, and the sources and methods of case ascertainment and control selection. Give the rationale for the choice of cases and controls  *Cross-sectional study*—Give the eligibility criteria, and the sources and methods of selection of participants | 4 | A cross-sectional design was used to recruit HPS and HCP in Saudi Arabia. |
|  |  | (*b*) *Cohort study*—For matched studies, give matching criteria and number of exposed and unexposed  *Case-control study*—For matched studies, give matching criteria and the number of controls per case |  | NA |
| Variables | 7 | Clearly define all outcomes, exposures, predictors, potential confounders, and effect modifiers. Give diagnostic criteria, if applicable | 6 | Simple linear regression analysis was conducted to determine predictors of professional identity (outcome) and sample characteristics (predictors). |
| Data sources/ measurement | 8* | For each variable of interest, give sources of data and details of methods of assessment (measurement). Describe comparability of assessment methods if there is more than one group | *5-6* | *Assessment of professional identity and Statistical analysis sections* |
| Bias | 9 | Describe any efforts to address potential sources of bias | 5 | Throughout the method section |
| Study size | 10 | Explain how the study size was arrived at | 4 | The minimum number of participants needed for this study was 184 HPS and 184 HCP based on expected number of healthcare students/workers in Saudi Arabia of 300,000, expected frequency of strong professional identity among 40% of the population, and 5% margin of error. Sample size calculations were done using Epi InfoTM (Epi Info 7.2.4.0, CDC, Atlanta). |

Continued on next page

| Quantitative variables | 11 | Explain how quantitative variables were handled in the analyses. If applicable, describe which groupings were chosen and why | 6 | HPS and HCP |
| --- | --- | --- | --- | --- |
| Statistical methods | 12 | (*a*) Describe all statistical methods, including those used to control for confounding | 6 | Statistical analysis section |
|  |  | (*b*) Describe any methods used to examine subgroups and interactions |  |  |
|  |  | (*c*) Explain how missing data were addressed |  | NA |
|  |  | (*d*) *Cohort study*—If applicable, explain how loss to follow-up was addressed  *Case-control study*—If applicable, explain how matching of cases and controls was addressed  *Cross-sectional study*—If applicable, describe analytical methods taking account of sampling strategy |  |  |
|  |  | (*e*) Describe any sensitivity analyses |  |  |
| Results | | | | |
| Participants | 13* | (a) Report numbers of individuals at each stage of study—eg numbers potentially eligible, examined for eligibility, confirmed eligible, included in the study, completing follow-up, and analysed | NA | Online survey with no exclusion criteria |
|  |  | (b) Give reasons for non-participation at each stage |  |  |
|  |  | (c) Consider use of a flow diagram |  |  |
| Descriptive data | 14* | (a) Give characteristics of study participants (eg demographic, clinical, social) and information on exposures and potential confounders | 6-7 | Table 1 |
|  |  | (b) Indicate number of participants with missing data for each variable of interest |  | NA |
|  |  | (c) *Cohort study*—Summarise follow-up time (eg, average and total amount) |  |  |
| Outcome data | 15* | *Cohort study*—Report numbers of outcome events or summary measures over time |  |  |
|  |  | *Case-control study—*Report numbers in each exposure category, or summary measures of exposure |  |  |
|  |  | *Cross-sectional study—*Report numbers of outcome events or summary measures | *8* | *Level of professional identity* |
| Main results | 16 | (*a*) Give unadjusted estimates and, if applicable, confounder-adjusted estimates and their precision (eg, 95% confidence interval). Make clear which confounders were adjusted for and why they were included |  | NA |
|  |  | (*b*) Report category boundaries when continuous variables were categorized |  | NA |
|  |  | (*c*) If relevant, consider translating estimates of relative risk into absolute risk for a meaningful time period |  | NA |

Continued on next page

| Other analyses | 17 | Report other analyses done—eg analyses of subgroups and interactions, and sensitivity analyses |  | NA |
| --- | --- | --- | --- | --- |
| Discussion | | | | |
| Key results | 18 | Summarise key results with reference to study objectives | 13 | The government of Saudi Arabia is making significant efforts to improve the quality of health education and overall healthcare system in the country. Yet, data concerning the professional identity of HPS and HCP are still lacking. We aimed in the present study to assess the level of professional identity in HPS and HCP and to investigate its predictors in Saudi Arabia. Findings of this study will serve as benchmark data that could guide decision-makers in the education system before and after transformation of the higher education system is applied to all public universities in Saudi Arabia. |
| Limitations | 19 | Discuss limitations of the study, taking into account sources of potential bias or imprecision. Discuss both direction and magnitude of any potential bias | 15 | The study is limited by the nature of the design used which does not allow the determination of causality and change in patterns. Additionally, due to the sampling method technique used to recruit participants, the generalizability of the study findings might be limited to participants who used social media applications frequently. However, the majority of the Saudi population, especially young and educated individuals, have Internet access and use social media applications. |
| Interpretation | 20 | Give a cautious overall interpretation of results considering objectives, limitations, multiplicity of analyses, results from similar studies, and other relevant evidence | 13-15 |  |
| Generalisability | 21 | Discuss the generalisability (external validity) of the study results | 15 | Additionally, due to the sampling method technique used to recruit participants, the generalizability of the study findings might be limited to participants who used social media applications frequently. However, the majority of the Saudi population, especially young and educated individuals, have Internet access and use social media applications. |
| Other information | |  | | |
| Funding | 22 | Give the source of funding and the role of the funders for the present study and, if applicable, for the original study on which the present article is based | NA | “The authors received no specific funding for this work.” Will be entered into the submission system as requested |

*Give information separately for cases and controls in case-control studies and, if applicable, for exposed and unexposed groups in cohort and cross-sectional studies.

**Note:** An Explanation and Elaboration article discusses each checklist item and gives methodological background and published examples of transparent reporting. The STROBE checklist is best used in conjunction with this article (freely available on the Web sites of PLoS Medicine at http://www.plosmedicine.org/, Annals of Internal Medicine at http://www.annals.org/, and Epidemiology at http://www.epidem.com/). Information on the STROBE Initiative is available at www.strobe-statement.org.
