# Supplementary material for: Exploring professional identity and its predictors in health profession students and healthcare practitioners in Saudi Arabia
Source: PLoS One. 2024 May 31;19(5):e0299356. doi: 10.1371/journal.pone.0299356 (PMC11142680; doi:10.1371/journal.pone.0299356)
Supplement: S1 Table — Numbers presented in table are frequencies (%). (DOCX) [file pone.0299356.s002.docx]

**Supporting information**

**S1 Table. Items included in the professional identity scale among health profession students and healthcare practitioners in Saudi Arabia.**

|  | **Strongly Agree** | **Agree** | **Neutral** | **Disagree** | **Strongly Disagree** |
| --- | --- | --- | --- | --- | --- |
| ***Health profession students (n= 185)*** | | | | | |
| 1. I feel that I am a member of this profession (specialization) | | | | | |
|  | 94 (50.8) | 60 (32.4) | 21 (11.4) | 5 (2.70) | 5 (2.70) |
| 2. I feel that I have strong relationships with professionals in the profession field (specialization) | | | | | |
|  | 33 (17.8) | 57 (30.8) | 53 (28.6) | 28 (15.1) | 14 (7.60) |
| 3. I feel ashamed to acknowledge that I belong to this profession (specialization) | | | | | |
|  | 5 (2.70) | 6 (3.20) | 15 (8.10) | 50 (27.0) | 109 (58.9) |
| 4. I find myself providing excuses for belonging to this profession (specialization) | | | | | |
|  | 6 (3.20) | 16 (8.60) | 23 (12.4) | 59 (31.9) | 81 (43.8) |
| 5. I try to hide that I am studying to be part of this profession (specialization) | | | | | |
|  | 4 (2.20) | 11 (5.90) | 2 (1.10) | 45 (24.3) | 123 (66.5) |
| 6. I am pleased to belong to this profession (specialization) | | | | | |
|  | 122 (65.9) | 44 (23.8) | 14 (7.60) | 5 (2.70) | 0 (0.00) |
| 7. I can identify positively with members of this profession (specialization) | | | | | |
|  | 79 (42.7) | 61 (33.0) | 34 (18.4) | 9 (4.90) | 2 (1.10) |
| 8. Being a member of this profession (specialization) is important to me | | | | | |
|  | 109 (58.9) | 55 (29.7) | 16 (8.60) | 4 (2.20) | 1 (0.50) |
| 9. I feel I share characteristics with other members of the profession (specialization) | | | | | |
|  | 47 (25.4) | 53 (28.6) | 60 (32.4) | 17 (9.20) | 8 (4.30) |
| ***Healthcare practitioners (n= 219)*** | | | | | |
| 1. I feel like a member of my profession, in which I work | | | | | |
|  | 114 (52.1) | 78 (35.6) | 22 (10.0) | 3 (1.40) | 2 (0.90) |
| 2. I feel that I have strong relationships with practitioners in my profession | | | | | |
|  | 93 (42.5) | 99 (45.2) | 16 (7.30) | 10 (4.60) | 1 (0.50) |
| 3. I feel ashamed to acknowledge that I belong to my profession | | | | | |
|  | 4 (1.80) | 5 (2.30) | 1 (0.50) | 54 (24.7) | 155 (70.8) |
| 4. I find myself providing excuses to belong to my profession | | | | | |
|  | 2 (0.90) | 7 (3.20) | 3 (1.40) | 75 (34.2) | 132 (60.3) |
| 5. I try to hide that I am part of this profession | | | | | |
|  | 1 (0.50) | 7 (3.20) | 9 (4.10) | 56 (25.6) | 146 (66.7) |
| 6. I am glad to belong to this profession | | | | | |
|  | 155 (70.8) | 50 (22.8) | 11 (5.00) | 1 (0.50) | 2 (0.90) |
| 7. I can identify positively with members of this profession | | | | | |
|  | 121 (55.3) | 82 (37.4) | 10 (4.60) | 3 (1.40) | 3 (1.40) |
| 8. Being a member of this profession is important to me | | | | | |
|  | 133 (60.7) | 77 (35.2) | 8 (3.70) | 1 (0.50) | 0 (0.00) |
| 9. I feel I share characteristics with other members of the profession | | | | | |
|  | 73 (33.3) | 100 (45.7) | 36 (16.4) | 8 (3.70) | 2 (0.90) |

Numbers presented in table are frequencies (%).
